# Wilms’ Tumor in a 37-Year-Old

**DOI:** 10.4021/jocmr377w

**Published:** 2010-07-20

**Authors:** Gowreeson Thevendran, Hugo A. Farne, Amir V. Kaisary

**Affiliations:** a Department of Surgery, St. Marys Hospital, London, UK; b Department of Urology, Royal Free Hospital, London, UK

## Abstract

Wilms’ tumor is rare in adults. Though the approach to diagnosis and treatment of adult Wilms’ tumor (AWT) is closely modeled on recommendations for childhood Wilms’ tumor, views differ on how aggressive the treatment should be. We report a case of a 37-year-old with Stage III favorable histology AWT. A radical nephrectomy was performed and the patient was due for chemotherapy. Recent advances, controversies and current recommendations in the treatment of AWT are discussed.

**Keywords:**

Adult; Wilms’ tumor; Kidney

## Introduction

Wilms’ tumor is a disease of young children accounting for approximately 8% of all childhood malignancies [1]. Although the incidence of Wilms’ tumor in adults is low, the exact number is unknown as a large number are either insufficiently documented or incorrectly diagnosed [2]. Jagazia and colleagues found a 9.2% incidence of adults among patients with Wilms’ tumor seen at one institution during a fourteen year tenure [3]. In the United Kingdom, the incidence of AWT is estimated at 6 per year, with only 1% of the total incidence of Wilms’ tumor (both adult and childrens) occurring in those above the age of fifteen [4].

Compared to their pediatric counterparts, adult Wilms’ tumor is assumed to have a poorer stage-for-stage prognosis [5]. Since 1979 however, with the use of combined modality treatment, studies have shown better improvement in response and survival, similar to those in children [2]. We report a case of Wilms’ tumor in a 37-year-old lady. She underwent a radical nephrectomy and is now receiving chemotherapy as per the National Wilms’ Tumor Study Group (NWTSG) protocol.

## Case Report

**Figure 1.**
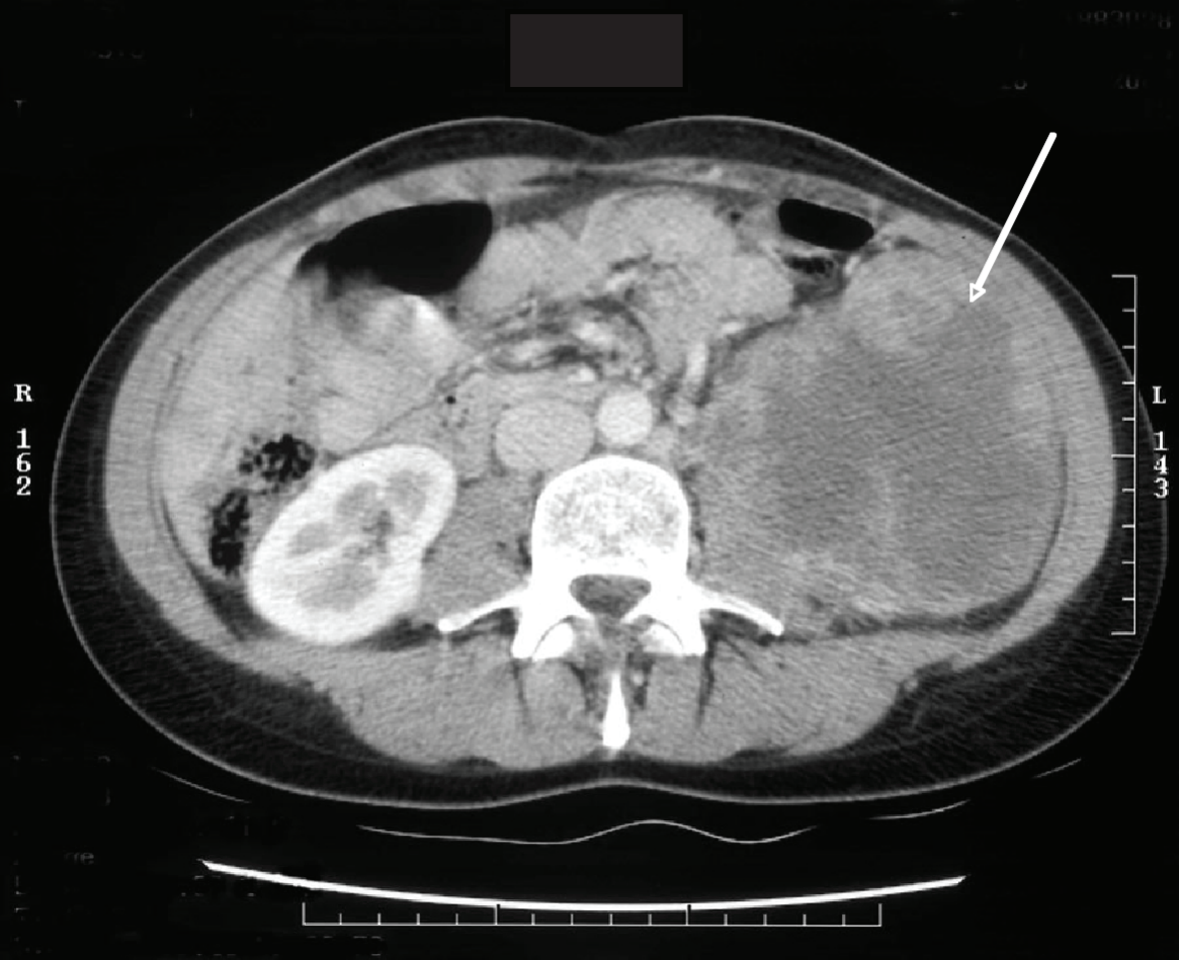
Post contrast CT demonstrating a 10 cm heterogeneously enhancing mass arising from the lower pole of the left kidney. It is centrally necrotic. The mass encroaches onto the left psoas and blocks the fat plane between itself and the left psoas muscle.

**Figure 2.**
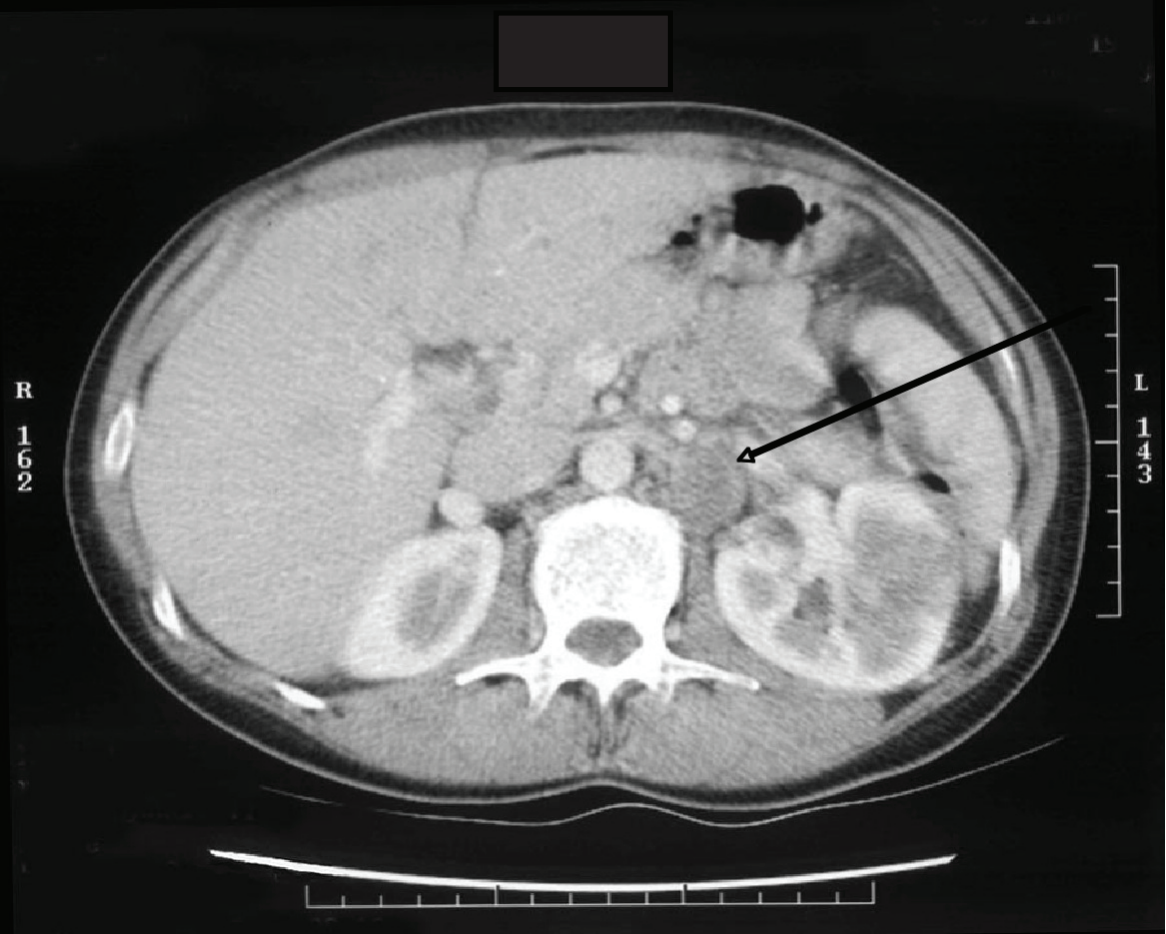
A 2.5 cm pathologically enhanced lymph node crossed by the left renal vein.

**Figure 3.**
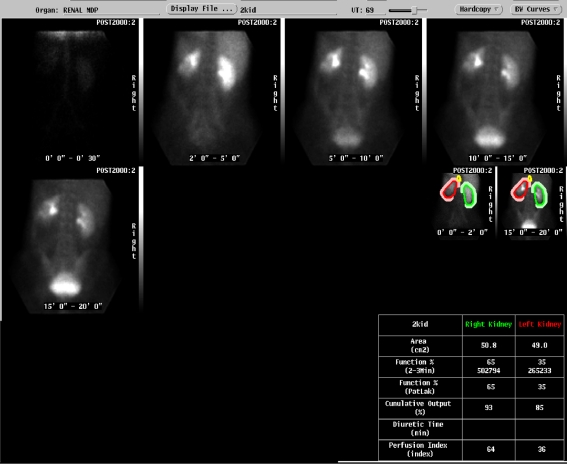
Absent function in inferior pole of the left kidney

A 37-year-old lady was referred by her general practitioner for a left flank mass. Enhanced Computed Tomography confirmed the presence of a 9.8 cm by 10.6 cm mass in the left flank region extending into the left iliac fossa (Fig. 1). The mass had encroached onto the left psoas major muscle with associated para aortic lymphadenopathy (Fig. 2). A staging bone scan demonstrated no evidence of metastatic disease. Renogram showed absent function of the inferior pole of the left kidney (Fig. 3) with a differential left kidney function of 35% of the total function.

**Figure 4.**
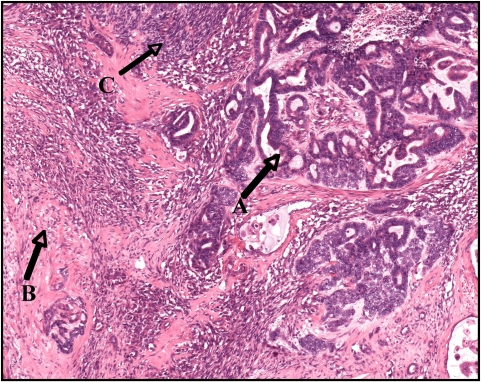
A triphasic adult Wilms’ tumor exhibiting a epithelial component (A), stromal component (B) and small areas of blastema (C)

**Figure 5.**
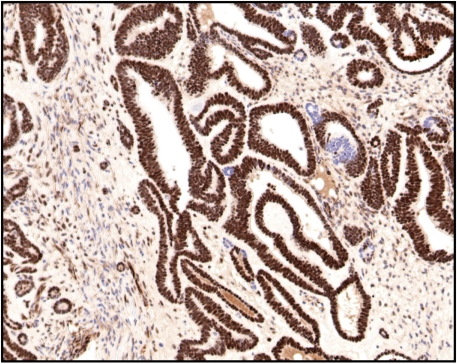
WT-1 staining showing strong nuclear positivity in the glandular component and weak nuclear positivity in the stromal component

In view of the high index of suspicion of malignancy, a left radical nephrectomy was performed. At surgery, a large mass was seen arising from the upper pole of the left kidney. Left para aortic lympadenopathy extending down to the left common iliac artery was noted. Subsequent histological examination of the nephrectomy specimen was reported as a large lobulated cream colored tumor composed of solid, necrotic and hemorrhagic areas. The tumor measured 95 × 95 × 142 mm and was situated in the upper pole and mid zone of the kidney. Microscopy revealed a triphasic tumor composed of epithelial, blastematous and stromal elements (Fig. 4, 5). There was extracapsular spread with up to five hilar lymph nodes involved. The diagnosis was a Stage III favorable histology Wilms’ tumor with clear resection margins.

## Discussion

Adult Wilms’ tumor is diagnosed in individuals who are older than 15 years [6]. Clinically, the tumor may be detected on abdominal palpation or as an incidental finding on a Computed Tomography or ultrasound scan performed for a different indication. Flank pain and hematuria are the commonest presenting complaints [6]. Compared to their pediatric counterparts, adult patients present more frequently with advanced clinical stages (metastases: 29% in adult Wilms’ tumor versus 10% in childhood Wilms’ tumor) [7].

Both in adults and children, Wilms’ tumors are three phase embryonic renal tumors made up of varying properties of blastemic, epithelial and mesenchymal structures. Of note, in adults, various undifferentiated tumors must be considered in the differential diagnoses especially when the tumor is predominantly monophasic [8]. From the genetic aspect, childhood Wilms’ tumor may occur as part of a number of pediatric syndromes. This is thought to involve alterations at multiple genetic loci including WT1 (chromosome 11p13), WT2 (chromosome 11p15) and WT3. Whether the adult disease is associated with a similar genetic aberration remains to be determined.

The major problem in the treatment of AWT is the lack of a defined therapeutic protocol [9]. The current treatment regime is modeled on the pediatric regimen recommended by the National Wilms’ Tumor Study Group. This consists of radical nephrectomy and adjuvant chemotherapy with or without radiotherapy, depending on the disease stage [10]. As for our patient, the current recommended treatment is a combination of 24 weeks of chemotherapy in addition to radiotherapy to the renal bed and para aortic lymph nodes.

Unfortunately, little information is available with respect to alternative treatment for adults where initial chemotherapy fails or the disease recurs. While the concern in children is preventing a second malignancy, in adults of child-bearing age like our patient, the issue of infertility associated with radiotherapy and subsequent compliance further complicates management. There is an ongoing debate with respect to the dose of radiotherapy in adult protocols and a clinical trial (SIOPWT) is looking at the possibility of avoiding both anthracyclines (which are cardiotoxic) and radiotherapy [4].

Looking to the future, various new modalities such as recombinant interferon alpha for the treatment of recurrent AWT are being explored. Perhaps, with the recommendations of the most recent UKW3 randomized trial, the question of neo adjuvant treatment versus surgery and the dilemma of reducing burden while maintaining efficacy in AWT will be addressed.
